# Epigenome-wide association study of level and change in cognitive abilities from midlife through late life

**DOI:** 10.1186/s13148-021-01075-9

**Published:** 2021-04-21

**Authors:** Ida K. Karlsson, Malin Ericsson, Yunzhang Wang, Juulia Jylhävä, Sara Hägg, Anna K. Dahl Aslan, Chandra A. Reynolds, Nancy L. Pedersen

**Affiliations:** 1 Department of Medical Epidemiology and Biostatistics, Karolinska Institutet, Stockholm, Sweden; 2 Institute of Gerontology and Aging Research Network – Jönköping (ARN-J), School of Health and Welfare, Jönköping University, Jönköping, Sweden; 3 Department of Health Sciences, School of Health Sciences and Welfare, University of Skövde, Skövde, Sweden; 4 Department of Psychology, University of California, Riverside, USA; 5 Department of Psychology, University of Southern California, Los Angeles, CA USA

**Keywords:** DNA methylation, EWAS, Cognition, Aging, Longitudinal

## Abstract

**Background:**

Epigenetic mechanisms are important in aging and may be involved in late-life changes in cognitive abilities. We conducted an epigenome-wide association study of leukocyte DNA methylation in relation to level and change in cognitive abilities, from midlife through late life in 535 Swedish twins.

**Results:**

Methylation levels were measured with the Infinium Human Methylation 450 K or Infinium MethylationEPIC array, and all sites passing quality control on both arrays were selected for analysis (*n* = 250,816). Empirical Bayes estimates of individual intercept (age 65), linear, and quadratic change were obtained from latent growth curve models of cognitive traits and used as outcomes in linear regression models. Significant sites (*p* < 2.4 × 10^–7^) were followed up in between-within twin pair models adjusting for familial confounding and full-growth modeling. We identified six significant associations between DNA methylation and level of cognitive abilities at age 65: cg18064256 (*PPP1R13L*) with processing speed and spatial ability; cg04549090 (*NRXN3*) with spatial ability; cg09988380 (*POGZ*), cg25651129 (-), and cg08011941 (*ENTPD8*) with working memory. The genes are involved in neuroinflammation, neuropsychiatric disorders, and ATP metabolism. Within-pair associations were approximately half that of between-pair associations across all sites. In full-growth curve models, associations between DNA methylation and cognitive level at age 65 were of small effect sizes, and associations between DNA methylation and longitudinal change in cognitive abilities of very small effect sizes.

**Conclusions:**

Leukocyte DNA methylation was associated with level, but not change in cognitive abilities. The associations were substantially attenuated in within-pair analyses, indicating they are influenced in part by genetic factors.

**Supplementary Information:**

The online version contains supplementary material available at 10.1186/s13148-021-01075-9.

## Background

The past decade has highlighted epigenetic influences, mechanisms regulating gene expression through reversible modifications, as major players in the aging process. The brain is one of the most affected organs, where substantial epigenetic changes result in decline in synaptic plasticity, memory, and learning [1]. These alterations may thus help explain the substantial individual variability seen in late-life cognitive ability, where some stay cognitively healthy and others suffer from debilitating cognitive decline [2–5]. DNA methylation is the most extensively studied epigenetic mechanism and refers to the addition of a methyl group to a cytosine located next to a guanine in the DNA, a so-called CpG site. Gene promotors are often rich in CpG sites, where hypermethylation is associated with downregulated gene expression and hypomethylation with upregulated expression [1]. DNA methylation is thus an important mechanism in tissue-specific transcriptional regulation, and studying differences and changes in DNA methylation can help us understand biological mechanisms underlying health and disease. However, the interpretation of associations is complicated as methylation levels at certain sites are influenced by genetic variants, so-called methylation quantitative trait loci (meQTLs) [6]. Twin and sibling designs present the opportunity to study DNA methylation while adjusting for the effects of meQTLs and examine the extent of familial confounding [7].

To the best of our knowledge, only three previous epigenome-wide association studies (EWAS) of cognitive abilities in population-based samples have been conducted. Marioni et al. [8] conducted an extensive meta-analysis across 11 cohorts (mean age 56–79), studying cross-sectional associations between blood DNA methylation and cognitive abilities in different domains. Starnawska et al. [9] studied blood DNA methylation in relation to level and 10-year linear change in general cognitive ability in 243 monozygotic twin pairs with a mean age of 70 at last measurement. Recently, the first EWAS of cognition in a Chinese sample was conducted by Wang et al., who studied blood DNA methylation and cognitive function in 30 monozygotic twin pairs with a median age of 52 years [10].

Considering the substantial changes occurring from mid- through late life in both cognitive abilities and the epigenetic landscape of the aging brain, age differences may have substantial effects on associations between DNA methylation and cognitive abilities. Therefore, we here aimed to add to previous work by studying epigenome-wide DNA methylation in association with both intercept level and longitudinal change in cognitive abilities (processing speed, verbal and spatial ability, episodic and working memory, and general cognitive ability). By utilizing a twin design, we also examined the presence and extent of genetic confounding. We first modeled the association between whole blood DNA methylation at CpG sites and cognitive abilities using empirical Bayes (EB) estimates to capture level and change in cognition, based on measures spanning up to 24 years from midlife through late life. Significant and suggestive associations were followed up in (1) analyses within twin pairs to investigate the extent of genetic and other familial confounding and (2) full latent growth-curve analyses of change in cognitive abilities. Significant sites were further characterized through follow-up analyses and look-up in previously published results from the same sample and in online tools to examine twin pair correlations in methylation levels, study longitudinal changes in methylation during aging, identify meQTLs, examine correlations between methylation levels in blood and brain, and study tissue expression of the relevant genes.

## Results

### Study population

The study was based on 535 individuals from the Swedish Adoption/Twin Study of Aging (SATSA) [11], a sub-study of the population-based Swedish Twin Registry (STR) [12]. Cognitive abilities covering processing speed, verbal ability, spatial ability, episodic memory, and working memory were tested during up to 10 in-person testing occasions. A measure of general cognitive ability was created based on all domains. Individuals diagnosed with dementia were censored from the time of diagnosis and onward. The mean number of cognitive assessments was 5.4 assessments (SD = 2.3, range 1–10) over an average of 15.0 years (SD = 7.6, range 0–27). Blood samples were collected from the third in-person testing occasion and onward, and DNA methylation measured from the first available blood sample was used in this study. The sample consisted of 313 (58.5%) women and 222 (41.5%) men, with a mean age of 61.8 (SD = 7.6, range 48–88) years at first participation and 68.2 (SD = 9.5, range 48–94) years at first blood sample. At the time of blood sampling, 95 individuals were current smokers (17.8%). The sample included 238 (82 monozygotic, 156 dizygotic) complete twin pairs. Baseline characteristics for the total sample and by DNA methylation array are presented in Additional file 1; Table S1. Individuals with DNA methylation measured on the 450 K array were significantly older at blood sample and first cognitive measure and had longer follow-up, but did not differ in level of cognitive abilities.

### EWAS of empirical Bayes estimates for level and change in cognitive abilities

In the first step of analyses, we performed an EWAS to identify epigenome-wide significant (threshold pre-defined at *p* < 2.4 × 10^–7^ [13]) and suggestive (threshold pre-defined at *p* < 10^–5^ [9]) signals.

As longitudinal models are computationally intense and hence not ideal for the EWAS setting, we first obtained EB estimates by applying linear and quadratic latent growth curve models [14] to each cognitive domain. Thus, individual measures of cognitive level at the intercept age and of the linear and quadratic change across time were obtained and used as separate outcomes in epigenome-wide analyses. To obtain more precise EB estimates, cognitive information across all in-person testing occasions was used, regardless of when methylation was measured. A quadratic model best fit the data for all domains except working memory, where the linear model showed the best fit. Intercept age was set at 65 for all domains except verbal ability where the intercept age 70 best fit the data (based on previous work [15]).

The epigenome-wide analyses were then modeled in linear regressions, with DNA methylation at each CpG site as the exposure and the EB estimates as separate outcomes. The models were adjusted for sex, age and smoking at time of blood sample, methylation array, number of testing waves with cognitive measures, and relatedness among the twins (see "Materials and methods" section for additional details). Estimates for linear and quadratic slopes were scaled to represent 10-year change.

Significant findings from the EWAS of DNA methylation and EB estimates of level and change in cognitive abilities are presented in Table 1 and suggestive findings in Additional file 2. In total, five CpG sites reached epigenome-wide significance, all with level of cognitive ability at the intercept age65: cg18064256 (*PPP1R13L*) with lower level of processing speed and spatial ability; cg04549090 (*NRXN3*) with higher level of spatial ability; cg08011941 (*ENTPD8*) and cg25651129 (-) with higher level and cg09988380 (*POGZ*) with lower level of working memory.

**Table 1:** Significant epigenome-wide associations of DNA methylation and empirical Bayes estimates for level and change in cognitive abilities

Cognitive domain and CpG site	Gene	Position^a^	Total sample *n* = 535 individuals			Between-pair effect *n* = 297 pairs			Within-pair effect *n* = 238 complete pairs		
			Estimate	SE	*p* value	Estimate	SE	*p* value	Estimate	SE	*p* value
Processing speed (intercept)											
cg18064256	*PPP1R13L*	19:45905621	− 1.77	0.31	1.55e−08	− 2.22	0.51	2.20e−05	− 1.14	0.34	9.42e−04
Spatial ability (intercept)											
cg04549090	*NRXN3*	14:79033036	1.95	0.36	1.23e−07	2.34	0.55	2.95e−05	1.02	0.37	6.55e−03
cg18064256	*PPP1R13L*	19:45905621	− 2.01	0.38	1.67e−07	− 2.15	0.56	1.45e−04	− 1.74	0.38	5.99e−06
Working memory (intercept)											
cg09988380	*POGZ*	1:151431765	− 2.11	0.40	1.88e−07	− 2.64	0.61	2.57e−05	− 1.04	0.48	0.03
cg25651129	*–*	8:11474056	1.89	0.35	1.34e−07	2.41	0.64	2.05e−04	1.21	0.44	6.28e−03
cg08011941	*ENTPD8*	9:140333139	2.00	0.35	2.67e−08	2.49	0.62	7.93e−05	1.01	0.47	0.03

Significant (*p* < 2.4 × 10^–7^) associations from epigenome-wide analyses of DNA methylation and level and change in processing speed, verbal and spatial ability, episodic and working memory, and general cognitive ability in the total sample, followed by results between and within twin pairs. Empirical Bayes estimates for level of cognitive ability at the intercept age (age 70 for verbal ability, 65 for all other domains) as well as 10-year linear and quadratic change were modeled as separate outcomes. Linear regression was applied to the total sample and between-within models to compare estimates between and within twin pairs. All models were adjusted for sex, age and smoking at the time of blood sample, methylation array, and number of testing waves with cognitive measures

^a^Genome Reference Consortium Human Build 37 (GRCh37)

Another 131 suggestive associations were identified (Additional file 2). Of note is that cg18064256 also showed a suggestive association with general cognitive ability at age 65 and 10-year linear change in processing speed and cg04549090 with level of general cognitive ability at age 65. Another 11 CpG sites showed suggestive associations with more than one cognitive domain or growth feature (Additional file 2). All sites with a significant or suggestive *p* value were carried forward to follow-up analyses of the respective cognitive domain.

DNA methylation levels at cg18064256 differed by DNA methylation array (Additional file 1, Table S1), but modeling the association separately for the 450 K and EPIC array demonstrated comparable effects (Processing speed. 450 K: *β* = − 1.93, standard error (SE) = 0.36; EPIC: *β* = − 1.35, SE = 0.62. Spatial ability. 450 K: *β* = − 2.13, SE = 0.46; EPIC: *β* = − 1.73, SE = 0.67).

### Between-within models of DNA methylation and empirical Bayes estimates for level and change in cognitive abilities

We applied between-within models [16], where the between-pair estimate represents the average effect in the population, while the within-pair estimate represents the effect after adjusting for factors shared within the twin pair. The latter is thus an estimate of the effect not attributable to shared genetic or other familial factors, and an attenuation of the association compared to the between-pair estimate indicates confounding by familial factors (e.g., meQTLs). As in the epigenome-wide analyses, DNA methylation at each CpG site was modeled as the exposure and EB estimates for cognitive level and change as the outcome, and the models adjusted for sex, age and smoking at time of blood sample, methylation array, and number of testing waves with cognitive measures.

Results from between-within models are presented in Table 1 for significant associations from the epigenome-wide analyses and in Additional file 2 for suggestive associations. All the significant associations presented were substantially reduced with, on average, halved regression estimates for the association between methylation on cognitive abilities within twin pairs compared to between pairs. This indicates that a relatively large part of the associations between DNA methylation and cognitive abilities at the significant sites are driven by genetic or other familial influences.

### Latent growth curve models of DNA methylation and level and change in cognitive abilities

Latent growth curve models [14] with age in decades as the timescale were fitted simultaneously with identified methylation sites to evaluate the trajectory features of cognitive abilities during late life, using cognitive data from the time of methylation measurement and onward. The intercept term here represents the level of cognitive ability at the intercept age (70 years for verbal ability, 65 years for all other domains), while the linear term represents the instantaneous linear rate of change at the intercept age, and the quadratic term the acceleration of change across age. As in the epigenome-wide analyses, the models were adjusted for sex, age and smoking at time of blood sample, methylation array, and relatedness among the twins. To evaluate the significance of the effect of methylation on level and change taken together, a likelihood ratio test was performed, comparing the model fit of the full model to that of a null model with only covariates and no methylation included. Standardized mean differences (Cohen’s d equivalents) in cognitive abilities by 1 SD higher DNA methylation were calculated for the intercept level and for change over 10 years from the intercept age (see "Materials and methods" section) [17].

**Fig. 1:**
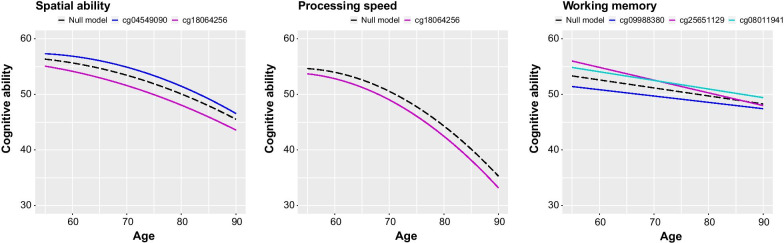
Longitudinal change in cognitive abilities. Estimated trajectories of change in cognitive abilities from the null models (without DNA methylation included; dashed lines) and with DNA methylation one standard deviation above the mean (solid lines) at CpG sites significantly associated with cognitive ability in EWAS analyses. Estimates were obtained from latent growth-curve models, with age (in decades) as the underlying timescale. Age was centered at 65 for all domains, and the models were further adjusted for sex, smoking, and methylation array

Growth features for each cognitive domain from a null model (without DNA methylation predictors) are presented in Additional file 1; Table S2. The intercept level ranged from 51.0 to 54.7, the linear slope from − 0.5 to − 3.4, and the quadratic slope from − 0.6 to − 1.4. The effects of DNA methylation on the intercept level, 10-year linear change, and 10-year quadratic change in cognitive abilities are presented in Table 2 (significant associations in the epigenome-wide analyses) and Additional file 3 (suggestive associations in the epigenome-wide analyses). Figure 1 visualizes the estimated growth trajectories with one SD higher methylation for the significant sites, alongside the estimated trajectories from the corresponding null model.

**Table 2:** The association between DNA methylation and longitudinal trajectories of cognitive abilities from latent growth-curve models

Cognitive domain and CpG site	CpG on intercept			CpG on linear change			CpG on quadratic change			LRT CpG	Cohen’s *d* equivalent	
	Estimate	SE	*p* value	Estimate	SE	*p* value	Estimate	SE	*p* value	*p* value	Intercept	Change 65–75
Processing speed												
cg18064256	− 1.35	0.32	2.90e−05	− 0.38	0.26	0.15	0.03	0.15	0.85	1.01e−05	0.14	0.04
Spatial ability												
cg04549090	1.37	0.35	1.11e−04	0.24	0.22	0.27	− 0.15	0.16	0.34	5.25e−04	0.14	0.01
cg18064256	− 1.71	0.35	1.41e−06	− 0.35	0.23	0.13	0.10	0.17	0.54	8.53e−07	0.17	0.03
Working memory												
cg09988380	− 1.61	0.37	1.48e−05	0.30	0.21	0.16	–	–	–	9.93e−05	0.16	0.03
cg25651129	1.83	0.35	2.80e−07	− 0.85	0.21	4.03e−05	–	–	–	2.63e−07	0.18	0.09
cg08011941	1.41	0.37	1.38e−04	− 0.11	0.22	0.61	–	–	–	4.22e−04	0.14	0.01

Mean cognitive level, 10-year linear change, and 10-year quadratic change in cognitive abilities in relation to DNA methylation at sites significant in EWAS analyses. Regression estimates, standard errors, and *p* values were obtained from full latent growth-curve models, simultaneously modeling the association between DNA methylation and intercept level, linear, and quadratic change in cognitive abilities. Age (in decades) was used as the underlying timescale, centered at age 65 for all domains. The models were further adjusted for sex, smoking at the time of blood sample, and methylation array. The model fit was compared to a null model not including DNA methylation to assess the significance of the effect of DNA methylation on cognitive level and change. Standardized mean differences (Cohen’s d equivalents) by 1 standard deviation higher DNA methylation at respective site was calculated for the intercept level and for 10-year change in cognitive abilities

*SE* standard error, *LRT* likelihood ratio test

One SD higher methylation level in cg18064256 (*PPP1R13L*) was associated with lower levels of processing speed and spatial ability at age 65, with a steeper linear decrease at age 65, but slightly less accelerating decrease. One SD higher methylation in cg04549090 (*NRXN3*) was associated with higher levels of spatial ability at age 65, a less steep rate of linear change at the same age, followed by a more accelerating decline. cg09988380 was associated with lower level of working memory at age 65 but a less steep linear rate of change. cg25651129 and cg08011941 were associated with higher level of working memory at age 65 but with a steeper linear decline. It should be noted that *p* values ranged between 2.80e−07 and 1.11e−4 for associations between CpG sites and intercept level, but were above 0.1 for associations between CpG sites and linear or quadratic change, with the exception of cg25651129 with linear change in working memory which had a *p* value of 4.03e−05.

The standardized effect sizes for the associations between DNA methylation and intercept cognitive level ranged between 0.14 and 0.18 (Table 2), thus considered of small magnitude [18]. The associations between DNA methylation and 10-year change from age 65 to 75 were of very small magnitude (0.01–0.09), but would reach larger magnitudes when cumulating over decades.

### Characterization of the CpG sites

To characterize longitudinal change in methylation at the five significant CpG sites and to identify meQTLs, we extracted results from a study by Wang and colleagues [19], which studied longitudinal change in methylation levels during aging and *cis*-meQTLs (within 1 million base pairs) in the SATSA sample. None of the five sites were significantly associated with age in the study by Wang et al., and we therefore did not further investigate longitudinal change in DNA methylation at these sites. No evidence of *cis*-meQTLs driving methylation was identified. We also performed lookup in the online mQTL database [6] (filtering on middle-age individuals) to identify *cis*- and *trans*-meQTLs driving methylation at the significant CpG sites. We here identified two potential (not meeting a strict *p* < 10^–14^ significance level [6]) *trans*-meQTLs: chrX:118976619:I is associated with methylation levels at cg04549090 (*p* = 4.77 × 10^–08^) and rs144382559 on chromosome 10 with cg08011941 (*p* = 9.53 × 10^–09^). To study whether these two meQTLs were associated with methylation level in the SATSA sample, the two SNPs were extracted from genotype data and modeled in linear regression models as predictors of DNA methylation at the relevant site and of the EB estimates for the relevant cognitive domain. The SNP on the X-chromosome was modeled separately for men and women. Neither of the SNPs were associated with methylation levels in this sample (chrX:118976619:I with cg04549090, *β* = − 0.02, *p* = 0.94 in women, *β* = 0.11, *p* = 0.57 in men; rs144382559 with cg08011941, *β* = − 0.08, *p* = 0.76), nor with cognitive level at age 65 (chrX:118976619:I with spatial ability, *β* = 26.98, *p* = 0.60 in women, *β* = − 16.31, *p* = 0.52 in men; rs144382559 with working memory, *β* = − 1.87, *p* = 0.33).

To further examine evidence of genetic factors driving DNA methylation levels at the significant sites, we compared twin pair correlations between monozygotic and dizygotic twin pairs and calculated the Falconer heritability, a crude measure of the unadjusted broad-sense heritability [20]. The Falconer heritability estimates ranged between 0.28 and 0.59 for cg04549090, cg18064256, and cg09988380, but were close to zero for cg25651129 and cg08011941 (Additional file 1; Table S3). This speaks against familial confounding driving the associations between two latter CpG sites and cognitive abilities. However, for all CpG sites, the 95% confidence intervals around the correlation estimates were wide, and the heritability estimates should be interpreted with caution.

To investigate whether DNA methylation in blood leukocytes is correlated with that in brain cells, we performed lookup in IMAGE-CpG [21], an online tool to compare methylation levels in blood and brain from live human tissues, and the Blood Brain DNA Methylation Comparison Tool [22], where methylation levels can be compared in blood and four different brain regions (prefrontal cortex, entorhinal cortex, superior temporal gyrus, and cerebellum) from postmortem samples. According to the Blood Brain DNA Methylation Comparison Tool [22], blood methylation levels of cg18064256 showed a moderate correlation with levels in the entorhinal cortex (*r* = 0.40, *p* = 5.4 × 10^–4^) and the superior temporal gyrus (*r* = 0.32, *p* = 5.7 × 10^–3^). None of the other CpG sites showed significant correlations between blood and brain methylation levels in either online tool.

To investigate expression of the genes across tissues, we performed additional lookup in the Human Protein Atlas [23] (available from http://www.proteinatlas.org). *PPP1R13L* (cg18064256) and *POGZ* (cg09988380) are both expressed in several tissues, including brain and blood where both show low brain region and blood cell-type specificity. *NRXN3* (cg04549090) is primarily expressed in the brain, with low region specificity, and blood where it is primarily expressed in basophils. *ENTPD8* (cg08011941) is mainly expressed in the intestines and is generally not expressed in brain or blood cells.

To confirm that the associations were not driven by cognitive decline in preclinical dementia, we tested whether DNA methylation at the significant sites differed by dementia status. No significant associations were identified (Additional file 1, Table S4).

## Discussion

In this EWAS of late-life level and change in cognitive abilities, we identified six significant associations. One site was associated with level of both spatial ability and processing speed at age 65, one with spatial ability at age 65, and three sites with level of working memory at age 65. By comparing the associations between and within twin pairs, we demonstrated a substantial effect of genetic or other familial influences, with on average 50% reduction of the effect when accounting for familial factors. In addition, 131 suggestive associations were identified, several of which were associated with more than one cognitive domain or trajectory feature. Follow-up in latent growth curve models revealed small effect sizes for associations between DNA methylation and cognitive level at the intercept age, and very small effect sizes for associations between DNA methylation and 10-year change in cognitive abilities. Follow-up analyses indicated that DNA methylation at these sites does not substantially change over time during late life and does not correlate between blood and brain to a large degree. Taken together, this may indicate that the findings represent systemic effects, either with long-term effects on cognitive level, or themselves affected by factors related to cognitive abilities (reverse causation).

Of note among the findings is cg18064256 which was significantly associated with both processing speed and spatial ability at age 65 and, in addition, identified in suggestive associations with linear change in processing speed and level of general cognitive ability at age 65. According to the Blood Brain DNA Methylation Comparison Tool [22], blood methylation levels at cg18064256 may be correlated with methylation in the entorhinal cortex and the superior temporal gyrus, mainly involved in memory [24] and processing of sound and speech [25], respectively. Lookup in the Human Protein Atlas [23] confirmed that the gene is expressed in the brain, across different regions. cg18064256 is located in the 5′ untranslated region of the *PPP1R13L* gene, encoding an inhibitor of NF-kappa-B (NFκB) and p53[26], and may be linked to cognitive abilities in late life through a role in neuroinflammation and neurodegeneration [27].

*NRXN3* (cg04549090), here significantly associated with spatial ability at age 65 and suggestively associations with general cognitive ability at age 65, is one of three members of the neurexin protein family, all involved in synaptic organization [28]. The gene is primarily expressed in the brain, and genetic variants in the neurexin genes are associated with a variety of neuropsychiatric disorders such as autism spectrum disorder and schizophrenia [28].

The CpGs associated with working memory reside in less well-characterized genes. De-novo mutations in *POGZ* (cg09988380) are causal for neuropsychiatric and neurodevelopmental disorders [29]. Expression analyses of the gene demonstrated that it is expressed in the brain throughout the developmental stage and indicated that the protein is involved in regulation of synaptic function and gene expression [29]. ENTPD8 (cg08011941) is a member of the E-NTPDase family, enzymes involved ATP metabolism, and, according to the Human Protein Atlas [23], expressed mainly in the intestine. While still poorly understood, there is evidence of purinergic signaling involved in neurological and degenerative disease, including AD, Parkinson’s disease, and multiple sclerosis [30].

None of the significant sites identified in this study were among the presented findings in the three previously published EWASs of cognitive abilities [8–10]. While that may be due to several factors, differences in age and cognitive measures between the study samples are likely to play a role. Out of the two CpG sites significantly (*p* < 1.7 × 10^–8^) associated with cross-sectional cognition in the work by Marioni et al. [8], cg12507869 was associated with phonemic verbal fluency, but showed no evidence of association with verbal ability in this sample (*p* = 0.31). It should be noted, however, that the tests are not directly comparable as the verbal fluency test considers executive functioning in addition to verbal ability, while the test used in this study does not. The other site cg21450381, associated with global cognitive function, did not pass QC in this sample and was not included in analyses. The findings presented (*p* < 10^–5^) by Starnawska et al. [9] of DNA methylation in relation to general cognitive ability in monozygotic twin pairs were either not replicated (*p* values ranging from 0.04 to 0.94) or did not pass QC in this sample. The study by Wang et al., studying DNA methylation and cognition in 30 Chinese monozygotic twin pairs, used a sequencing-based rather than chip-based method [10]. Out of the 574,708 CpG sites analyzed, 28 were associated with cognitive functioning at *p* < 10^–4^. These were located in 11 known genes, none of which were significantly associated with cognitive abilities in the present study. This may be due to difference in genetic background and applied methods, in addition to differences in age and cognitive measures.

This study was based on a well-established twin sample with longitudinal and robust measures of cognitive abilities. The lack of replication of results from previous studies is a limitation, but also highlights the need for further work in the area. The presence of meQTLs complicates epigenetic studies, as almost 20% of the variance in DNA methylation may be driven by genetic factors, the majority acting through distant *trans* effects [6]. No evidence of *cis*-meQTLs was identified in the SATSA sample or in the mQTL database [6, 19], but we did identify two potential *trans*-meQTLs. These were not associated with methylation at the relevant sites or with the cognitive domains in this sample and hence do not account for the genetic confounding identified in the between-within analyses of the sites. This highlights the value of twin designs in methylation studies, as they offer a natural way to adjust for genetic influences such as meQTLs. However, twin designs also carry some limitations that need considering. While the within-pair estimates are by default adjusted for factors shared by the twins, they may still be influenced by biases from non-shared confounders and measurement error [31]. It should be noted that twin correlations confirmed genetic influences on three of the five significant sites, but were close to zero for two sites. While studying methylation in blood cells in relation to cognitive abilities is not ideal, conducting methylation studies of longitudinal changes in cognition is not feasible using neuronal tissue due to its inaccessibility. We compared DNA methylation levels in blood and brain using online tools, which, despite limited sample sizes, are highly valuable resources to understand how DNA methylation in blood samples may relate to processes in the brain. Only cg18064256 showed evidence of correlation between blood and brain and only in the Blood Brain DNA Methylation Comparison Tool [22]. This tool examines correlations between premortem blood samples and postmortem brain tissue from four different regions in 71–75 individuals. IMAGE-CpG [21] examines correlations in DNA methylation between blood samples and neuronal tissue (adjusted for cell counts) collected from 27 living individuals, aged 5–61. It should be noted that, as substantial epigenetic changes occur in the brain during aging [1], correlations between blood and brain methylation may differ across age as well as between pre- and postmortem samples. In addition, DNA methylation is not only cell type specific but also highly specific to brain regions and neuronal populations [1]. Thus, while the limited evidence of correlation between blood and brain DNA methylation in the current study indicates that systemic effects drive the associations, it is still plausible that correlations were not captured by the online tools, and that the associations are in fact driven by epigenetic processes in the brain.

## Conclusions

This EWAS of cognitive level and change during late life contributes to the growing body of evidence highlighting the role of DNA methylation in cognitive aging. We identified six associations between blood DNA methylation and level of processing speed, spatial ability, and working memory at age 65. The genes harboring these sites implicate processes involved in regulation of neuroinflammation, synaptic organization and functioning, ATP metabolism, and neuropsychiatric disorders. All associations were substantially reduced within twin pairs, indicating they are partly, but not completely, driven by familial factors. Follow-up in longitudinal analyses confirmed that DNA methylation at the CpG sites is predominantly associated with cognitive level, rather than change. Further characterization of the CpG sites indicated that DNA methylation levels do not substantially change during late life, and do not correlate between blood and brain to a large degree. Taken together, this may indicate that the findings represent systemic effects, either with long-term effects on cognitive level, or the result of reverse causation, themselves affected by factors related to cognitive abilities.

## Material and methods

### Study population

SATSA has been described in detail previously [11], but briefly, it is a longitudinal study of same-sex twin pairs who were reared apart, matched to a sample of twin pairs reared together. The study consists of up to 10 in-person testing occasions performed at approximately 3-year intervals between 1984 and 2014. The testing occasions included a health examination, cognitive tests, an interview, and collection of blood samples. A total of 859 individuals participated in at least one testing occasion, of whom 535 had information on DNA methylation from whole blood and were included in this study.

All participants provided informed consent, and the study was approved by the Regional Ethics board at Karolinska Institutet, Stockholm.

### Cognitive measures

At each testing occasion, cognitive tests were performed covering four domains: processing speed (Symbol Digit and Figure Identification (Form A) tests), verbal (WAIS Information subtest and Synonyms) and spatial abilities (Block Design and Card Rotations (Form A) tests), and memory which was divided into episodic (Thurstone’s Picture Memory Task) and working memory (Digit Span, forward and backward) [32, 33]. A measure of general cognitive ability was created based on principal component analysis of all individual tests comprising the four domains, standardized relative to means and variances at the first testing occasion [15]. Only non-demented individuals were included in the current analyses, such that individuals who developed dementia contributed data only before dementia diagnosis. Prior to analyses, all measures were transformed into T-scores with mean 50 and a standard deviation of 10, scaled to the first in-person testing occasion.

### DNA methylation measurements

DNA methylation was available from blood samples collected during the third, fifth, sixth, eighth, ninth, and tenth in-person testing occasions. Not all individuals participated in each testing occasion, and we therefore used DNA methylation data from the first available time point.

Extracted DNA was first bisulfate converted with the EZ-96 DNA MagPrep methylation kit (Zymo Research Corp., Orange, CA, USA) and hybridized onto the Infinium Human Methylation 450 K Bead Chip (*n* = 385), or the Infinium MethylationEPIC BeadChip (*n* = 150, both from Illumina Inc., San Diego, CA, USA). The raw data were pre-processed using a rigorous quality control pipeline (described in detail previously [19]). Samples with poor correlation to genotype controls or with the wrong predicted sex based on signal ratio from the sex chromosomes were removed, as were probes overlapping a SNP site, residing on sex chromosomes, or with detection *p* value above 0.05. R was used for processing the data, applying methylumi.noob [34] for background correction, wateRmelon.dasen [35] for normalization, and the ComBat function in the sva package [36] to adjust for batch effects (slide). Cell counts were not available from the samples, and the normalized data were therefore corrected for cellular compositions using the Houseman method [37] based on a blood cell reference panel [38]. Methylation levels at each site were transformed to *M* values (the logit-2 transformed ratios of methylated to unmethylated probe intensity) for their better statistical properties [39], and the *M* values were further standardized for easier interpretation.

CpG sites were selected for analyses based on the following criteria: present on both methylation arrays; passing the QC on both methylation arrays; less than 15% difference in mean methylation between the two arrays (*n* = 4540 removed). This resulted in a total of 250,816 CpG sites.

### Covariate and genotype data

Information about sex and date of birth was available in the STR data, and age at each in-person testing occasion calculated. Smoking is known to substantially affect DNA methylation [40], and we therefore adjusted for smoking status at time of blood sample (current smoking or not smoking, using self-reported data from the testing occasion).

SATSA participants were genotyped on Illumina PsychArray (Illumina Inc., San Diego, CA, USA), and the data imputed against the 1000 Genomes Project phase 1 version 3 reference panel [41].

### Statistical analyses

#### Selection of significant and suggestive thresholds

There has been some debate regarding *p* value threshold for epigenome-wide significance, as there is still limited knowledge of how methylation across CpG sites is correlated. As we here study different, but highly correlated, outcomes, we selected the epigenome-wide *p* < 2.4 × 10^–7^ threshold suggested for Illumina 450 K data by Saffari et al. [13], based on permutation methods. While we analyzed substantially fewer CpG sites (~ 250 K) and the threshold in this case is close to that of a Bonferroni adjustment, considered too strict in EWAS studies, it was calculated taking correlation of methylation across sites into account, and in our case also leaves room for considering the different outcomes. However, as this threshold may be too stringent, we defined a suggestive threshold at *p* < 10^–5^ (used in the study by Starnawska et al. [9]) and present those results in Additional files.

#### EWAS of empirical Bayes estimates for level and change in cognitive abilities

EB estimates for each cognitive domain were obtained by applying latent growth curve models [14] in SAS 9.4 (PROC MIXED) with twin pair ID as random effect. Linear and quadratic models were applied to all cognitive domains and EB estimates obtained from the best fitting model [according to the Akaike information criterion (AIC)] [42]. For each cognitive domain, the EB estimates for intercept level, linear, and quadratic change (based on linear and quadratic age, the latter not included for working memory) were then saved and used as separate outcomes in epigenome-wide analysis.

The epigenome-wide analyses were done by applying linear regression models using the lm function in R 3.5.2. DNA methylation at each CpG site was modeled as the exposure and the EB estimates as separate outcomes. To account for difference in the number of testing waves with cognitive measures, the contribution of each individual was weighted by the inverse SE of the EB estimate. Sex, age and smoking at time of blood sample, and methylation array (450 K or EPIC) were included as covariates in the models. As the inclusion of related individuals violates the assumption of independent observations, robust SEs were used to correct for relatedness among the twins. Age was treated as a continuous variable, sex and methylation array as categorical variables, and smoking as a binary variable.

All sites with a significant or suggestive *p* value were carried forward to follow-up analyses of respective cognitive domain.

#### Between-within models of DNA methylation and empirical Bayes estimates for level and change in cognitive abilities

Between-within models [16] were applied using linear mixed models in SAS 9.4 (PROC MIXED, SAS Inc., Cary NC) to study the extent of genetic and other familial influences. In this design, both the twin-pair mean methylation (between-pair estimate) and the individual deviation from the twin-pair mean (within-pair estimate) are modeled as fixed-effect predictors of cognitive abilities. As in the epigenome-wide association analyses, DNA methylation at each CpG site was modeled as the exposure and EB estimates for cognitive level and change as the outcome. Individuals were weighted by the individual inverse SEs of the EB estimates, and twin pair IDs were included as a random effect. The models were adjusted for sex, age and smoking at time of blood sample, and methylation array as above.

#### Latent growth curve models of DNA methylation and level and change in cognitive abilities

Latent growth curve models with age in decades as the timescale were then fitted. The models included fixed effects, linear, and quadratic trends, with age centered at 65 years for each cognitive outcome, except for verbal ability where age was centered at 70 [15] and working memory where quadratic trends were not included. Methylation level at baseline was modeled as a fixed effect on the level of cognitive abilities at the intercept age and in interaction with age to investigate the effect of baseline methylation levels on longitudinal cognitive trajectories. To account for relatedness between the twins, individual IDs nested within twin pair IDs were modeled as random effects. Random effects on the intercept and linear age were included on both the individual and pair ID level in all models, except for working memory where the model did not support random effects on linear age on the pair ID level. Models for processing speed, verbal and spatial ability (except two suggestive CpGs), and general cognitive ability also supported random effects on quadratic age on the individual ID level, but not on the pair ID level. The model for episodic memory and for two CpGs on spatial ability (cg08972756 and cg18833907) did not support random effects on quadratic change on either individual or pair ID level, and the effect of baseline methylation on quadratic change could not be studied. The models were adjusted for sex, smoking status at baseline (time of blood sample), and methylation array (included as fixed effects). Sex and methylation array were converted to binary variables (sex: 0 = male, 1 = female; methylation array 0 = EPIC, 1 = 450 K) to facilitate model conversion. The estimates for intercept, linear, and quadratic slope thus represent cognitive level and 10-year change (at age 65, except for verbal ability where intercept was set at age 70) for a non-smoking male whose methylation levels were analyzed on the EPIC chip. The significance of the effect of methylation on level and change taken together was evaluated in likelihood ratio tests with 2 (models with methylation effects on intercept and linear change) or 3 (models with methylation effects on intercept, linear, and quadratic change) degrees of freedom, comparing the model fit (− 2 log likelihood) of the full model described above to that of a null model with only covariates and not methylation included. Cohen’s *d *[18] equivalents of effect sizes for the standardized mean difference in cognitive level at the intercept age by 1 SD higher DNA methylation were calculated as:

$$d_{{{\text{intercept}}}} = \beta_{{{\text{intercept}}}} /{\text{SD}}$$

Standardized mean differences in 10-year change in cognitive abilities (from the intercept age) by 1 SD higher DNA methylation were calculated according to Feingold’s formula for time-varying effect sizes for quadratic change [17]:

$$d_{T} = \, (\beta_{{{\text{linear}}}} *T + \beta_{{{\text{quadratic}}}} + T^{2} ) \, /{\text{SD}}$$

where SD = 10 (as cognitive measures were transformed into *T*-scores), and duration is indexed in the formula as *T*, and *T* = 1 was used to calculate mean differences for 10-year change (as age in decades was the model timescale). For linear models, the quadratic term was omitted from the equation.

To visualize the difference in cognitive level and change, the estimated trajectories from the null models (without DNA methylation) and from the methylation models were plotted with the ggplot2 [43] package in R.

## Supplementary Information

**Additional file 1: Table S1-S4.** Table S1: Descriptive statistics of the total study sample and stratified by DNA methylation array. Table S2: Null model for intercept level, linear change, and quadratic change in cognitive abilities. Table S3: Comparison of twin-pair correlation between monozygotic and dizygotic twin-pairs. Table S4: Differences in DNA methylation in relation to dementia status.

**Additional file 2.** Suggestive epigenome-wide associations of DNA methylation and level and change in cognitive abilities. Suggestive (p < 10^–5^) associations from epigenome-wide analyses of DNA methylation and level and change in processing speed, verbal and spatial ability, episodic and working memory, and general cognitive ability, in the total sample, followed by results between and within twin pairs.

**Additional file 3.** Associations between DNA methylation at suggestive sites and longitudinal trajectories of cognitive abilities. Cognitive level, linear change, and quadratic change in cognitive abilities in relation to DNA methylation at suggestive sites from EWAS.

## Data Availability

The datasets supporting the conclusions of this article are available at National Archive of Computerized Data on Aging under accession number ICPSR 3843 (phenotypic data; https://www.icpsr.umich.edu/web/NACDA/studies/3843) and the EMBL‐EBI repository under accession number E‐MTAB‐7309 (DNA methylation data; https://www.ebi.ac.uk/arrayexpress/experiments/E-MTAB-7309). All codes used to generate analysis data and for conducting analyses are available at https://github.com/ik-karlsson/EWAScognition.
