# Supplementary material for: Epigenome-wide association study of level and change in cognitive abilities from midlife through late life
Source: Clin Epigenetics. 2021 Apr 21;13:85. doi: 10.1186/s13148-021-01075-9 (PMC8061224; doi:10.1186/s13148-021-01075-9)
Supplement: Supplementary file 1 — Additional file 1: Table S1-S4. Table S1: Descriptive statistics of the total study sample and stratified by DNA methylation array. Table S2: Null model for intercept level, linear change, and quadratic change in cognitive abilities. Table S3: Comparison of twin-pair correlation between monozygotic and dizygotic twin-pairs. Table S4: Differences in DNA methylation in relation to dementia status. [file 13148_2021_1075_MOESM1_ESM.pdf]

Supplementary section:

Epigenome-wide association study of level and change in cognitive abilities from midlife through late-life

Ida K. Karlsson, Malin Ericsson, Yunzhang Wang, Juulia Jylhävä, Sara Hägg, Anna K. Dahl Aslan, Chandra A. Reynolds, Nancy L. Pedersen

Contact: ida.karlsson@ki.se

Table S1: Descriptive statistics of the total study sample, and stratified by DNA methylation array

Table S2: Null model for intercept level, linear change, and quadratic change in cognitive abilities

Table S3: Comparison of twin-pair correlation between monozygotic and dizygotic twin-pairs

Table S4: Differences in DNA methylation in relation to dementia status

Table S1: Descriptive statistics of the total study sample, and stratified by DNA methylation array

	Total sample	By DNA methylation array		
		**450K**	**EPIC**	**P-value**
Number of individuals, N	535	385	150	--
Number of individuals with ≥3 measures, N	478	352	126	--
Women, N (%)	313 (58.50)	230 (59.74)	83 (55.33)	0.41
Smoker at blood sample, N (%)	95 (17.76)	65 (16.88)	30 (20.00)	0.47
Age at blood sample, mean (SD)	68.19 (9.49)	68.93 (9.62)	66.3 (8.88)	<0.01
cg04549090, mean (SD)	-5.00 (0.59)	-5 (0.62)	-4.99 (0.53)	0.92
cg18064256, mean (SD)	0.67 (0.40)	0.64 (0.41)	0.72 (0.38)	0.04
cg09988380, mean (SD)	-5.38 (0.23)	-5.37 (0.23)	-5.4 (0.21)	0.16
cg25651129, mean (SD)	2.31 (0.42)	2.3 (0.44)	2.32 (0.37)	0.56
cg08011941, mean (SD)	2.46 (0.43)	2.47 (0.45)	2.46 (0.37)	0.83
Age at first cognitive measure, mean (SD)	61.81 (7.55)	62.22 (7.79)	60.78 (6.79)	0.04
Follow-up time, cognition, mean (SD)	14.95 (7.62)	15.74 (7.53)	12.91 (7.48)	<0.01
Number of cognitive measures, mean (SD)	5.37 (2.35)	5.64 (2.35)	4.7 (2.20)	<0.01
Processing speed, mean (SD)	53.37 (9.87)	53.42 (10.24)	53.23 (8.85)	0.83
Verbal ability, mean (SD)	52.14 (9.30)	52.03 (9.52)	52.44 (8.73)	0.64
Spatial ability, mean (SD)	52.83 (10.30)	52.96 (10.71)	52.5 (9.20)	0.62
Episodic memory, mean (SD)	52.24 (9.70)	52.6 (9.55)	51.31 (10.07)	0.18
Working memory, mean (SD)	51.61 (10.28)	51.47 (10.33)	51.96 (10.17)	0.62
General cognitive ability, mean (SD)	53.21 (9.95)	53.25 (10.37)	53.11 (8.75)	0.88

Note. Descriptive statistics for the total sample and stratified by DNA methylation array. The total number of individuals represent the total analysis sample, and the number of individuals with at least 3 cognitive measures those contributing to estimates of longitudinal change (linear and quadratic slope). Cognitive measures represent level at the first available measurement occasion. Statistical significance of differences by DNA methylation array was tested by χ^2 test (sex and smoking status) or two sample t-test (all other variables).

Table S2: Null model for intercept level, linear change, and quadratic change in cognitive abilities

	Intercept			Linear slope			Quadratic slope			Correlations			
										Individual level			Pair level
	Beta	SE	P-value	Beta	SE	P-value	Beta	SE	P-value	Icpt-slope	Icpt-slope^2	Slope-slope^2	Icpt-slope
**Processing speed**	52.61	1.02	1.14E-130	-3.43	0.28	2.75E-26	-1.40	0.16	2.40E-17	0.55	-0.67	-0.76	0.23
**Verbal ability**	54.47	1.03	7.59E-133	-0.73	0.14	3.85E-07	-1.05	0.10	2.30E-21	0.11	-0.2	0.41	0.07
**Spatial ability**	54.70	1.07	4.19E-129	-2.22	0.24	8.09E-18	-0.58	0.17	6.12E-04	0.28	-0.74	-0.15	0.43
**Episodic memory**	51.00	0.98	9.98E-132	-0.50	0.29	9.05E-02	-1.16	0.17	4.51E-12	0.16	-	-	-0.65
**Working memory**	51.88	1.00	5.00E-132	-1.44	0.23	3.51E-10	-	-	-	-0.25	-	-	-
**General cognition**	54.27	1.10	2.50E-125	-1.99	0.19	9.74E-21	-1.15	0.14	2.98E-15	0.35	-0.51	-0.15	0.50

Cognitive level, 10-year linear change, and 10-year quadratic change in processing speed, verbal and spatial ability, episodic and working memory, and general cognition. Beta values, standard errors, p-values, and correlations across growth features were obtained from latent growth-curve models, with age (in decades) as the underlying time scale. Age was centered at 65 for all domains except verbal ability, where age 70 was used. The models were further adjusted for sex, and smoking (included as fixed effects). Random effects on intercept and linear age were included on both the individual and twin pair level for all domains, except for linear age on the pair level for working memory. Random effects on quadratic age were included on the individual level for all domains except episodic and working memory.

Icpt: intercept; SE: standard error; slope^2: quadratic slope.

Table S3: Comparison of twin-pair correlation between monozygotic and dizygotic twin-pairs

CpG	rMZ (95% CI)	rDZ (95% CI)	Falconer heritability
cg04549090	0.18 (-0.04-0.38)	0.04 (-0.12-0.19)	0.28*
cg18064256	0.36 (0.15-0.53)	0.06 (-0.10-0.21)	0.59*
cg09988380	0.30 (0.09-0.48)	0.13 (-0.02-0.28)	0.33
cg25651129	-0.02 (-0.23-0.20)	-0.03 (-0.19-0.13)	0.02
cg08011941	0.03 (-0.19-0.24)	0.04 (-0.12-0.20)	-0.02

Twin-pair correlations and 95% confidence intervals of DNA methylation at five CpG site for monozygotic (rMZ) and dizygotic (rDZ) twin pairs. The Falconer heritability was calculated with the Falconer formula as 2 x (rMZ - rDZ) and is a measure of the unadjusted broad-sense heritability.

*Potential non-additive/dominance genetic effects, as indicated by the low rDZ and high rMZ

Reference: Falconer DS, Mackay TFC. Introduction to quantitative genetics. 4th ed. ed. Harlow: Longman; 1996

Table S4: Differences in DNA methylation in relation to dementia status

CpG	Estimate	SE	P-value
cg04549090	0.08	0.12	0.49
cg18064256	0.02	0.13	0.89
cg09988380	-0.01	0.12	0.96
cg25651129	0.05	0.15	0.73
cg08011941	0.02	0.13	0.91

Differences in DNA methylation by dementia status (dementia diagnosis during follow-up, versus cognitively intact throughout follow-up) were tested in linear regression models, adjusted for age, sex, and smoking at the time of blood sample, and methylation array.
